# devfOLD: a toolbox for designing age-specific fNIRS channel placement

**DOI:** 10.1117/1.NPh.8.4.045003

**Published:** 2021-12-06

**Authors:** Xiaoxue Fu, John E. Richards

**Affiliations:** University of South Carolina, Department of Psychology, Columbia, South Carolina, United States

**Keywords:** near-infrared light spectroscopy, channel placement, Monte Carlo simulation, toolbox, infant, development

## Abstract

**Significance:** Near-infrared spectroscopy (NIRS) is a noninvasive technique that uses scalp-placed sensors to measure cerebral hemoglobin concentration. Commercial NIRS instruments do not allow for whole-head coverage and do not intrinsically indicate which brain areas generate the NIRS signal. Hence, the challenge is to design source–detector channel arrangement that maximizes sensitivity to a given brain region of interest (ROI). Existing methods for optimizing channel placement design have been developed using adult head models. Thus, they have limited utility for developmental research.

**Aim:** We aim to build an application from an existing toolbox (fOLD) that guides NIRS channel configuration based on age group, stereotaxic atlas, and ROI (devfOLD).

**Approach:** The devfOLD provides NIRS channel-to-ROI specificity computed using photon propagation simulation with realistic head models from infant, child, and adult age groups.

**Results**: Cortical locations and user-specified specificity cut-off values influence the between-age consistency and differences in the ROI-to-channel correspondence among the example infant and adult age groups.

**Conclusions**: The study highlights the importance of incorporating age-specific head models for optimizing NIRS channel configurations. The devfOLD toolbox is publicly shared and compatible with multiple operating systems.

## 1 Introduction

Near-infrared spectroscopy (NIRS) is a noninvasive technique that uses scalp-placed sensors to measure cerebral hemoglobin concentration. Functional NIRS (fNIRS) is a noninvasive neuroimaging technique that measures event-evoked changes in cerebral blood oxygenation. A key advantage of the technology is its ease of use with participants of different ages. Hence, NIRS enables researchers to track neurodevelopment from infancy to adulthood.^1^^–^^3^

Changes in the intensity of light that flows from a source to a detector optode may be used to recover hemoglobin concentration changes in the cortex. The signals recorded at scalp channel locations contain the effect of hemodynamic activities from the brain as well as extracerebral tissues.^4^ The distance between source and detector influences the depth of the tissue sensitivity for a source to detector channel.^5^^,^^6^ The source–detector channel positions determine the sensitivity of the channel to the underlying cortex and the cortical location being sampled.^6^ Hence, optode placement is crucial for ensuring that the channels measure hemoglobin concentration changes in the cortical region of interest (ROI). Whereas there is a veridical relation between the optode placement and the underlying cortical areas generating the NIRS signal, it is a challenge to make informed decisions on the optimal channel arrangement for measuring a specific ROI.

Existing tools for designing optode placement have been developed based on adult head models. However, the scalp-location-to-ROI correspondence^7^^,^^8^ and channel-sensitivity profiles^5^ change during infancy through childhood and adulthood. The current study builds from an existing toolbox (fNIRS optodes’ location decider; fOLD^9^) to facilitate the age-specific design of channel placement in NIRS research for infants, children, and adults. Our developmental fNIRS optodes location decider (devfOLD) extends the fOLD to developmental populations.

The design of NIRS channel arrangement relies on understanding the mapping between scalp positions and ROIs. One approach is spatial scalp projection. This uses a spatial projection from a scalp location to the cortical surface to identify the corresponding ROI(s).^10^ The method is based on a systematic mapping between 10–10 scalp electrode locations and the underlying cortical regions.^11^^,^^12^ The projection-based method has been implemented in optode placement design based on the 10–10 system^13^ or an optimization method without the constraints of the standard electrode locations.^14^ Scalp projection typically defines the NIRS “channel” location as a point equidistant from the source and detector optodes. It does not consider the interaction between near-infrared light and the optical properties of the head tissue. It is likely that the source–detector channel is sensitive to more ROIs than the region(s) mapped to the channel location using vertical spatial projection.

Photon transport simulations provide an effective method for quantifying the sensitivity of a given source–detector channel to the underlying cortical region. Photon simulations model how photons propagate through the heterogeneous tissue structures of the head.^15^ Channel sensitivity can be quantified as the product of the fluence distribution at the source and the detector location (PMDF,^16^ S-D channel DOT,^5^ or three-point Green’s function^6^). The computation provides a sensitivity profile for each NIRS source–detector channel to represent the extent to which the channel can detect changes in optical properties in the given region of tissue.

The photon transport simulation method has been used in several channel placement optimization methods. These methods vary in the levels of burden required from researchers and participants. Machado et al.^17^ proposed a personalized approach that estimates sensitivity profiles using the individual’s own MRI scan to identify the channel layout for an ROI. The MRI-dependent method also requires customized optode placement or NIRS holder construction for individual participants. These additional requirements in MRI data collection limit the accessibility of NIRS, particularly for developmental studies. The AtlasViewer toolbox^18^ supports the design of optode configurations with the option of using the default adult head model (Colin27),^19^ subject-specific MRIs,^20^ or age-appropriate average templates.^21^ First, users specify a preliminary optode configuration and register the optode positions to the scalp surface of a head model. Next, the photon transport simulation is performed to estimate the sensitivity distribution of each channel. The user can then evaluate whether there is sufficient overlap between the sensitivity distribution of the channel to the underlying ROI(s). The optode placement can be optimized accordingly in an iterative process.^20^ However, it requires the additional procedure of digitizing scalp optode locations,^20^ expertise for evaluating channel-to-ROI sensitivity, and manual optimization of optode configuration.^16^ The Array Designer^16^ provided an optimization method that eliminates the burden of optode location digitization and manual optimization. It allows users to specify the ROI, the number of available sources and detectors, and the range of channel separation distance. It implements an algorithm that determines the source–detector arrangement in the 10–2.5 system with maximized sensitivity to the ROI as well as optimized coverage for the ROI based on the user inputs. As commercial fNIRS systems typically offer holders with 10–10 or 10–20 indications (e.g., NIRx and artinis), users may need to custom-make holders to fit the suggested channel configuration. The fOLD toolbox^9^ estimated sensitivity (calculated as “S-D channel DOT”) of a set of preselected source–detector pairs from the 10–10 and 10–5 systems. Channel-to-ROI specificity, defined as the sensitivity of a given channel to ROIs relative to the whole brain, was calculated and integrated into the toolbox. The fOLD toolbox provides a user-friendly tool that enables researchers to determine channels with high specificity for measuring their selected ROI.

One important limitation of the existing tools for NIRS optode arrangement designs is their limited applicability to a wide range of age groups. The fOLD toolbox^9^ and Array Designer^16^ used head models constructed from MRIs from a single (Colin27)^19^ or a group of adults (MNI-ICBM152).^22^ It is likely that channel sensitivity estimates computed on adult templates do not apply to developmental samples. Studies that examined computed S-D channel DOT for 10–10 channel locations using realistic head models have found both consistencies and inconsistencies in the channel-to-ROI mapping across infant age groups^7^ as well as among infant, child, and adult age groups.^8^ For example, Fu and Richards^8^ found that there was consistency in some channel-to-ROI mappings across infant, child, and adult age groups but divergence in others. For example, channel positions at F7, F8, F5, F6, FC5, and FC6 were sensitive to the inferior frontal gyrus for all age groups. Alternatively, the pattern of sensitive channel positions for the postcentral gyrus varied across age groups.

There are also age differences in how channel sensitivity profiles vary with source–detector separation distances. Channel sensitivity changes as a function of separation distances. As the source–detector separation increases, the source–detector fluence distribution covers wider tissue regions, extends deeper into the cortex, and decreases in fluence strength.^6^^,^^23^ Fu and Richards^5^ showed that there were also age-group differences in the shape of sensitivity profiles across the infant, child, and adult age groups. For example, at 35 mm of separation (close to the median distance of channels used in the fOLD toolbox^9^), the child and adult groups had relatively high peak and rapid decline of fluence strength as penetration depth increased. In contrast, age groups from 6 months to 2 years showed smaller peak and more gradual decline. Therefore, these findings underscore the importance of using age-specific head models to estimate channel sensitivity to ROIs. The extant channel placement optimization methods developed using adult head models cannot be readily applied to infants and children.

This study provides an age-specific tool for guiding NIRS channel arrangement in infant, child, and adult age groups. We selected the fOLD toolbox^9^ as a platform for incorporating age-specific functionalities because it provides a more convenient design tool for users compared to the AtlasViewer^18^ or Array Designer.^16^ It does not require digitizing optode locations or customizing NIRS holders. We present the devfOLD, a user-friendly tool that uses the same computation methods as the fOLD toolbox. The devfOLD provides three types of channel-to-ROI specificity estimates for each age group: (1) averaged age group estimations calculated by averaging specificity values from individual head models in each age group and averaging across participants within an age; (2) specificity estimates from an individual participant head model; and (3) specificity estimates from average age-appropriate templates. We also expanded the selections of developmentally appropriate stereotaxic atlas parcellations for ROIs. We did not intend to add additional features to the fOLD toolbox nor provide an optimization algorithm (as the Array Designer). Rather, the devfOLD toolbox provides an accessible tool for visualizing channel-to-ROI correspondence for groups of infants and developmental populations.

## 2 Materials and Methods

### 2.1 Participants

Data for the devfOLD toolbox were computed for the infancy period (2 weeks, 1 month, 2 months, 3 to 10.5 months with 1.5-month interval, 12 months, 15 months, 18 months, and 24 months), child ages (4 and 12 years), and young adults (20 to 24 years). Participants’ MRIs were obtained from open-access databases and a local scanning facility. All studies had institutional review board approval and informed consent for participants. Details of the full sample can be found in Table S1 in the Supplemental Material and our previous study.^8^ The current study aimed to compare channel-to-ROI mapping between infant and adult age groups. Hence, estimations for the 3-month, 6-month, and 20- to 24-year groups were presented in the toolbox as examples. Data collected from 238 participants at 3 months ($N_{\text{Total}}=38$; $N_{\text{Male}}=17$), 6 months ($N_{\text{Total}}=74$; $N_{\text{Male}}=39$), and 20 to 24 years ($N_{\text{Total}}=126$; $N_{\text{Male}}=50$) of age. The MRIs were collected from open-access databases and a local scanning facility. The 3-month sample was obtained from the Baby Connectome Project^24^ (N=24) or collected at the McCausland Center of Brain Imaging (MCBI) (N=14). The 6-month sample was from the Infant Brain Imaging Study^25^ (N=60) and the MCBI or collaborative studies (N=14). All 20- to 24-year data were collected from the MCBI (N=95) or collaborative studies (N=31). All studies had institutional review board approval and informed consent/assent. All parents and children completed written consent/assent and received monetary compensation for their participation. The University of South Carolina Institutional Review Board approved data collection at the MCBI and the use of data from all open-access databases.

### 2.2 MRI Sequences

The present study utilized T1-weighted (T1W) and T2-weighted (T2W) scans from each collection site. Details of the MRI acquisition protocols have been described in literatures on the Neurodevelopmental MRI Database.^26^^–^^31^ All MRIs were converted to NIFTI compressed format with 32-bit floating point resolution. Bias-field inhomogeneity correction (N4 algorithm) was performed on the extracted T1-weighted images.^32^^,^^33^

### 2.3 MRI Preprocessing and Segmentation

The brains were extracted from the whole-head MRI volume in a procedure adapted from the FSL VBM pipeline.^34^ The T1W volume for each participant was registered to an age-appropriate average MRI template. The average MRI templates are an unbiased representation of the average for the age group. The average templates came from the neurodevelopmental MRI database.^29^^–^^31^ The brain from the average template was transformed into the participant MRI space and used a mask on the head volume. The extracted masked data were then used with the FSL brain extraction tool program.^35^^,^^36^ Each brain was visually inspected and manually modified if necessary.

**Fig. 1:**
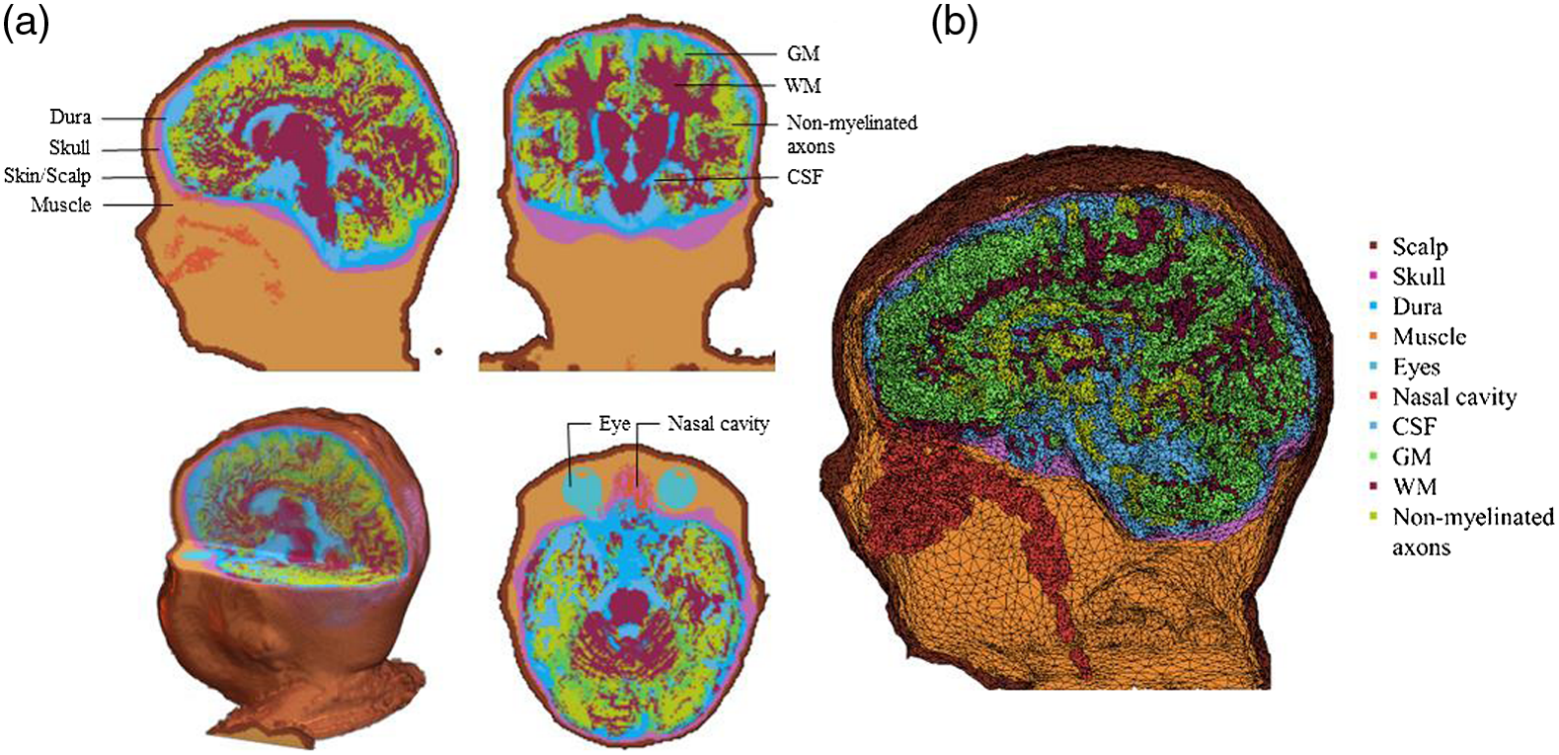
Segmented head MRI volumes for a 6-month infant MRI. The figure is adapted from Ref. 41. (a) The segmented head model. (b) The segmented head model with dense FE mesh (segmented FE mesh).

We performed finite element method segmentation. Each head MRI volume was segmented into 9 or 10 media types: gray matter (GM), white matter (WM), cerebrospinal fluid (CSF), nonmyelinated axons (NMA), other brain matter, skin, skull, air, eyes, and other inside skull materials. The FSL FAST procedure^37^ was used to segment the T1-weighted images into GM, WM, or other matter (OM). The tentative GM/WM classification from the initial step is problematic for infants at 12 months of age or younger whose brain lacks myelination.^30^^,^^38^ For infants in these age groups, the pattern of GM/WM in the 2-year-old average MRI template was used as a probability map to distinguish nonmyelinated tissues that should later be myelinated axons (WM), those that were GM or other nonmyelinated tissues. The CSF was removed from the materials from the FAST procedure, with the remainder defined as GM, WM, NMA, or other inside skull materials. The BETSURF procedure^35^^,^^36^ was used with the extracted brain, T1W and T2W volumes, to identify skull and scalp regions. The nasal cavity and eyes were identified manually using MRIcron.^39^^,^^40^ Finally, any OM inside the head volume not defined as above was defined as “other inside skull material.” This generally was in the region of the neck and consisted primarily of muscle and secondarily of spinal bone. Figure 1(a) shows a three-dimensional (3D) rendering of the T1W volume from a 6-month-old infant with a cutout revealing the segmented MRI volume. The realistic head model represents the geometry of the head and allows us to differentiate optical properties of different tissue types.

We additionally conducted a boundary element method (BEM) segmentation that segmented the MRI volume into scalp, skull, CSF, and brain. This was performed to replicate the sensitivity computations for the fOLD toolbox.^9^ In addition to the individual MRIs, age-appropriate average templates were constructed from the individual MRIs of an age range. The age-appropriate average templates had segmenting constructed from averaging of the individual MRIs for GM, WM, CSF, nasal cavity, and from the age-appropriate average head for skin, skull, eyes, and air.

### 2.4 Mesh Generation

A finite element (FE) tetrahedral mesh was constructed from the segmented head MRI volume. Figure 1(b) shows meshes that were produced using the iso2mesh toolbox with CGAL 3.6 mesh program (“v2m” function^42^). Tetrahedral meshes accurately represent the boundaries of complex 3D volumetric tissues and increase the accuracy in modeling photon propagation in complex mediums such as the head and brain.^43^ A mesh was generated for each segmented head MRI volume. Figure 1(b) shows an example of the FE mesh. The FE volumetric meshes have nodes that represent the voxel locations for the tetrahedra, a four-element matrix representing the corners of each tetrahedron, and a vector representing the media type from the segmented head MRI volume. The “segmented FE mesh” was used for MCX^44^ to find a segment element that was closest to an electrode position.

### 2.5 Scalp Locations

**Fig. 2:**
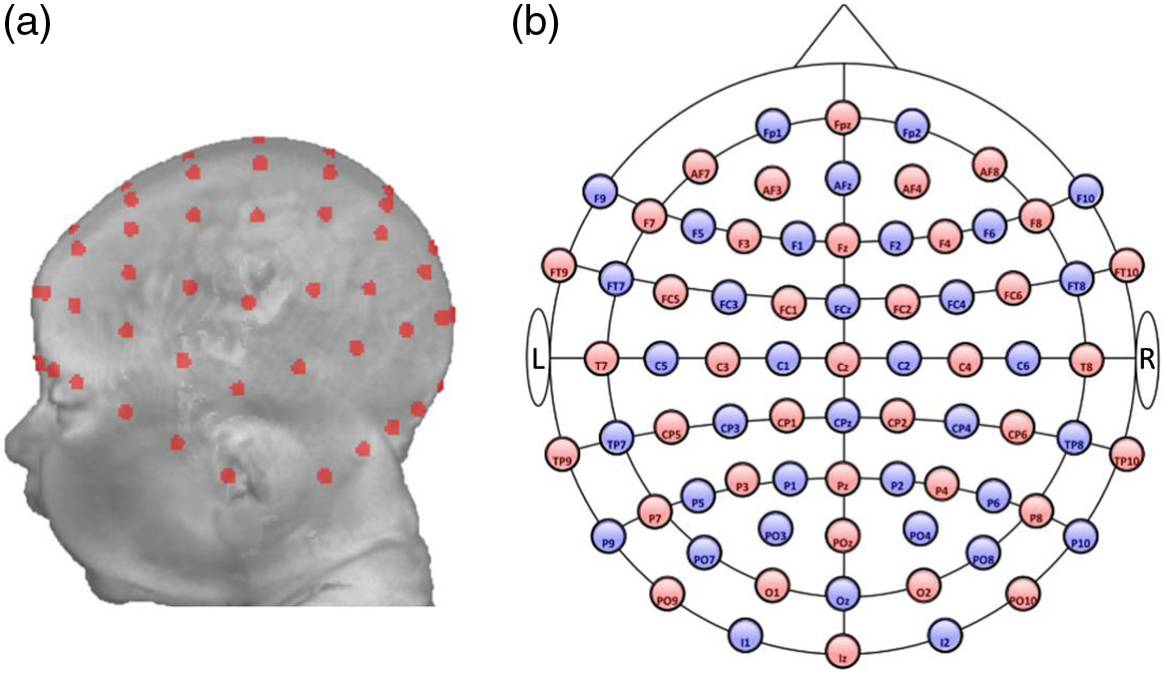
Optode and channel locations. (a) 10–10 virtual electrode placement on a 3-month individual head model. (b) A two-dimensional (2D) layout of the 10–10 systems. Sources are labeled in red, and detectors are denoted in blue. They form 130 source–detector channels.

The locations for the 10–10 electrode systems and positions of source–detector channels were constructed on each head MRI volume. We simulated 81 virtual electrode positions based on the “unambiguously illustrated 10–10 system.”^45^ Figure 2(a) shows the 10–10 electrode placement on an individual head MRI volume. Details for constructing the 10–10 locations are described in Refs. 8 and 46. The electrode-defined optode positions were constructed based on the fOLD toolbox.^9^ We selected 74 of the 10–10 locations to form 38 sources and 36 neighboring detectors. The 10–10 locations NZ, N1, AF9, T9, N2, AF10, and T10 were not used. There were a total of 130 source–detector channels. Figure 2(b) shows the optode locations. The source–detector pairings for all channels are presented in Table S2 in the Supplemental Material.

### 2.6 Brain Stereotaxic Atlases

Six stereotaxic atlases were constructed for each MRI volume to delineate anatomical regions that can be used to determine the sensitivity of source–detector channels to the ROI. The atlases were constructed on individual MRI volumes.^8^^,^^27^^,^^47^ First, the lobar atlas was an automatically constructed lobar atlas that identified the major cortical lobes (e.g., frontal), some sublobar cortical (e.g., fusiform gyrus), subcortical (e.g., striatum), cerebellum, and brainstem. Second, the Hammers atlas^48^ consists of 84 areas defined from the cortex, subcortical, brainstem, and cerebellum. Third, the Brainnetome connectivity atlas provides a microanatomical parcellation of 210 cortical and 36 subcortical subregions based on the local structural connectivity.^49^ Fourth, the Desikan–Killiany–Tourville (DKT) atlas is implemented in the parcellation algorithm of the FreeSurfer^50^ and the Infant FreeSurfer.^51^^,^^52^ The adult version contains 68 cortical, 38 noncortical/subcortical, and 4 WM regions. The infant version contains 68 cortical, 26 noncortical/subcortical, and 2 WM regions. Fifth, the LONI Probabilistic Brain Atlas (LPBA40)^53^ contains 56 areas from the cortical and subcortical regions, brainstem, and cerebellum. Sixth, we constructed the AAL3 atlas that is implemented in the SPM12 software.^54^ It includes 166 cortical and subcortical regions with additional anterior cingulate, thalamus, and brain nuclei parcellations compared to AAL2.

### 2.7 Photon Migration Simulations

Photon transport simulations were performed to estimate channel sensitivity to the cortex. Photon migration through head tissues for each FE segmented MRI volume was modeled using the Monte Carlo eXtreme package (MCX)^44^ that employs a GPU-accelerated, voxel-based Monte Carlo simulation algorithm. We launched $10^{8}\;\text{photons}$ from all 10–5 positions for the time window of 0 to 5 ns, though we only used the 74 10–10 positions used in the fOLD toolbox. The fluence resolution was 50 time gates.^9^ The wavelength was set at 690 nm. The optical properties of the 10 tissue types were set in accordance with previous studies.^55^^–^^58^ These are displayed in Table S3 in the Supplemental Material. The output of the simulations included the sum photon flux (or fluence) for each voxel. The number ranged from zero (i.e., all photons exited the media) to the total number of injected photons (i.e., all photons were absorbed).

**Fig. 3:**
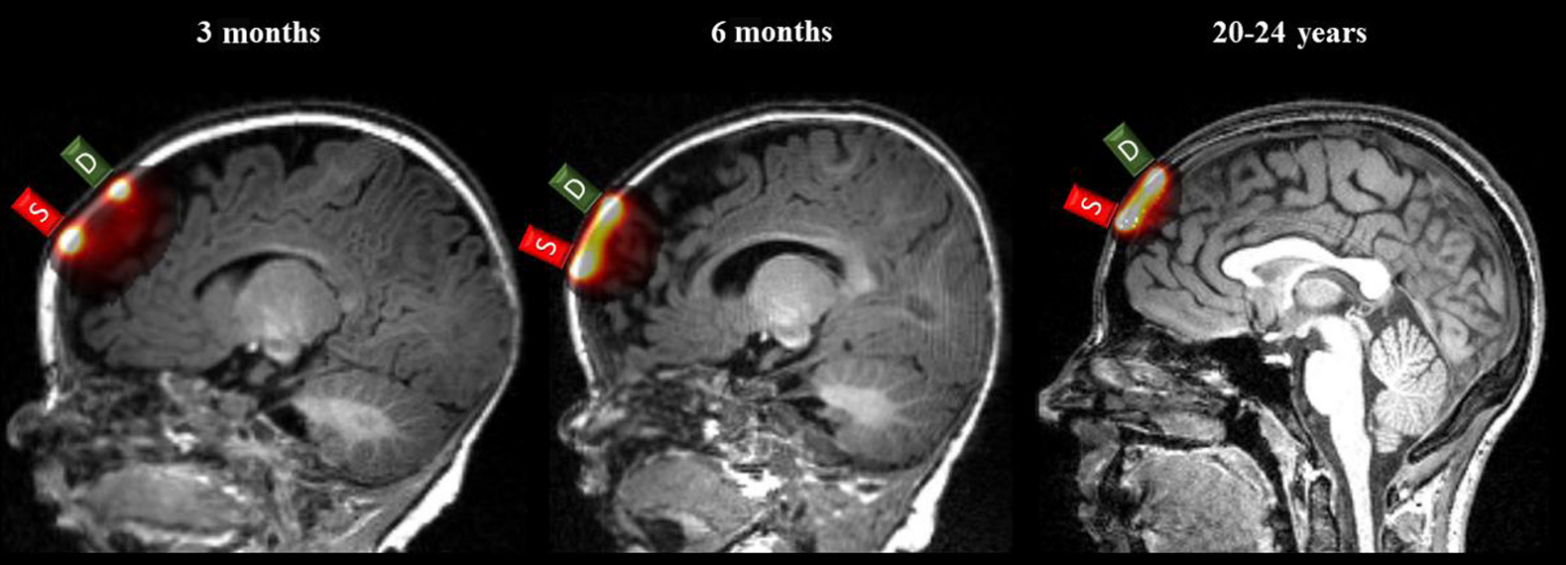
The output of photon migration simulation of sensitivity normalization for a single channel. The normalized sensitivity (normSens) result for channel FPz-AFz is displayed on an individual MRI volume from each age group. The color scale ranged from $10^{-5}$ to $3\times 10^{-4}$.

The output of the photon migration simulations was used to compute the sensitivity for each source–detector channel. The computation for the normalized channel sensitivity (normSens) presented in Eq. (1) was replicated from the fOLD toolbox.^9^ We divided the fluence absorbed at the optode by the total number of all the photons absorbed inside the head media for each source and detector position to obtain ${\text{fluence}}_{s}$ and ${\text{fluence}}_{d}$ at the voxel level. This normalization was consistent with prior studies.^9^^,^^18^^,^^59^ Next, we calculated the voxelwise normalized channel sensitivity (normSens^4^^,^^9^) for each of the 130 channels. This was calculated as the voxelwise (i) product of the normalized fluence at the source (${\text{fluence}}_{s}$) and detector position (${\text{fluence}}_{d}$)^4^^,^^59^ then divided by the sum of sensitivity of all voxels inside the MRI volume. The normSens value for each voxel at the channel location represents a percentage of sensitivity relative to the whole MRI volume. Figure 3 displays the normSens for the channel formed by source FPz and detector AFz on an individual MRI volume for each age group:

$$\text{normSens}(\mathrm{ch})=\overset{\text{head}}{\underset{i}{\sum }}\frac{{\text{fluence}}_{s}(\mathrm{ch},i)\times {\text{fluence}}_{d}(\mathrm{ch},i)}{{\text{fluence}}_{\text{sum}}(\mathrm{ch},i)}.$$ (1)

The voxelwise normSens data were used in subsequent computations for the key variable for interest: channel-to-ROI specificity. The specificity values were calculated for individual head models that were constructed from a participant’s MRI or an average template for a given age group. The individual-level computations were based on the fOLD toolbox.^9^ The group-level mean specificity for each channel was calculated by averaging the specificity values across participants from a given age group. The three types of computations were carried out to showcase that the channel placement design can be based on channel-to-ROI specificity estimations obtained using head models from (1) all individuals from the age group of interest, (2) a participant’s head model, and (3) an age-appropriate average template.

### 2.8 Individual-Level Calculations of Specificity and Channel Information

We first calculated the sensitivity of each channel to the brain (brainSens) for individual MRI volumes and average age-appropriate template. Equation (2) shows that brainSens was computed by summing normSens from all voxels (j) categorized as the brain in the BEM segmentation.^9^ The value for each brain voxel at a given channel represents the corrected sensitivity in respect to the whole MRI volume. We also calculated the channel sensitivity to the scalp, skull, and CSF by summing normSens from all voxels identified for each of the tissue types:

$$\text{brainSens}(\mathrm{ch})=\overset{\text{brain}}{\underset{j}{\sum }}\text{normSens}(\mathrm{ch},j).$$ (2)

We calculated the specificity (%) and uncorrected sensitivity of each channel to ROI parcellations in each atlas. The calculation for the channel specificity was identical to the fOLD toolbox.^9^ For each channel position, the normSens value at the voxel within an ROI (k) was divided by the brainSens value, and the output was summed across all voxels in the ROI and multiplied by 100. Hence, Eq. (3) illustrates that the specificity of the given channel represents the percentage of sensitivity to the given ROI out of the total sensitivity of the channel to the participant’s brain. Sensitivity of voxels that were not assigned to an ROI but had brainSens values was calculated as specificity to “Brain_Outside.”^9^ Hence, the corrected specificity to the ROIs and “Brain_Outside” that were measurable to the channel summed to 100. The corrected specificity and channel information for each individual MRI volume and average age-appropriate template can be displayed in the toolbox. The uncorrected sensitivity was calculated by summing normSens of the voxels belonging to the given ROI. The value represents the sensitivity to the ROI in respect to the MRI volume. Hence, the uncorrected sensitivity values for the ROIs that were measurable to the channel, “Brain_Outside,” scalp, skull, and CSF summed to 1. The uncorrected values were used to compute age-group-level channel specificity (see the following section):

$${\text{Specificity}}_{\mathrm{ROI}}(\mathrm{ch})=100\times \overset{\mathrm{ROI}}{\underset{k}{\sum }}\frac{\text{normSens}(\mathrm{ch},k)}{\text{brainSens}(\mathrm{ch})}.$$ (3)

We computed the coordinates (x,y,z) and the source–detector separation distance for each channel registered to the individual MRI volume to provide additional channel information. Equation (4) illustrates that the coordinate for each channel (${\text{Coordinate}}_{x,y,z}$) was calculated as the weighted mean of the coordinates of the brain voxels (j). The weight is the normSens normalized by the brainSens of the channel. Hence, ${\text{Coordinate}}_{x,y,z}$ denotes a sensitivity-weighted location on the head model for the given channel. The source–detector separation (mm) was calculated as the distance from the source to the detector that formed the channel:

$${\text{Coordinate}}_{x,y,z}(\mathrm{ch})=\overset{\text{brain}}{\underset{j}{\sum }}\frac{{\text{Coordinate}}_{x,y,z}(j)\times \text{normSens}(\mathrm{ch},j)}{\text{brainSens}(\mathrm{ch})}.$$ (4)

### 2.9 Group-Level Calculations of Specificity and Channel Information

**Fig. 4:**
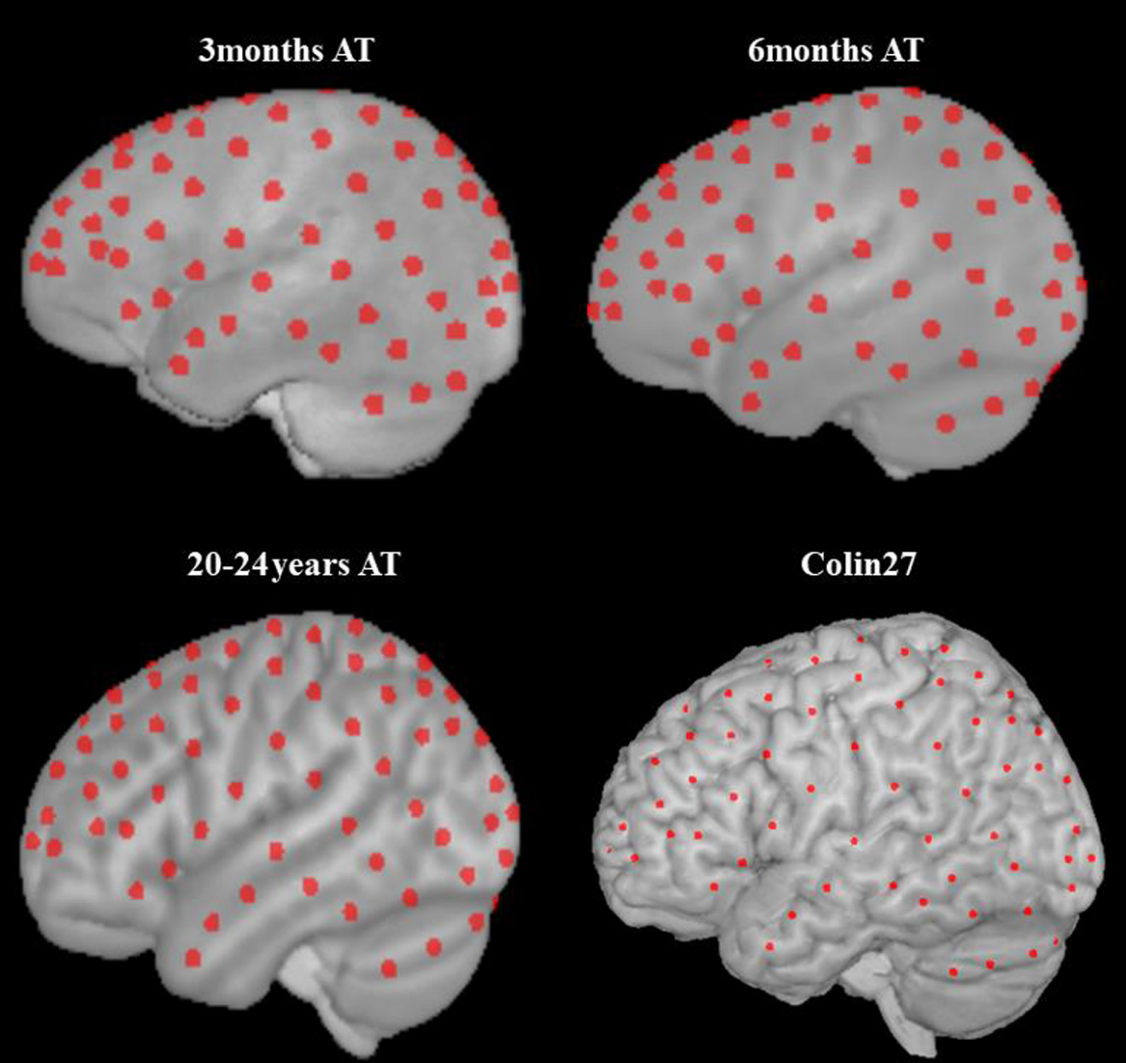
Group-level channel locations. The channel coordinates were weighted by the normalized sensitivity (normSens) in respect to the sensitivity to the brain (brainSens) of the given channel. The coordinates computed in the current study were in the age-matched average template space. The channel coordinates calculated in Ref. 9’s study were in the Colin27 (displayed here) or the SPM12 head model space.

We computed the specificity of a given channel to the ROIs, channel coordinates, and mean source–detector separation distances for each age group. Specificity of the given channel was calculated by averaging the uncorrected sensitivity values for each ROI across participants from the age group (e.g., 3 months, 6 months, and 20 to 24 years). The mean for the ROI was then divided by the sum of averaged sensitivity for all ROIs. Hence, the specificity to all ROIs summed up to 100 for the channel. We coregistered the channel locations in the individual MRI space to the age-matched average template using the “Coherent Point Drift” program in MATLAB (CPD).^60^ We obtained the affine transformation between the 10–10 electrode locations for the individual MRI space and those for the average template. The channel coordinates of the individual MRI were then transformed into the average template coordinate space. The group-level channel coordinates were calculated by averaging the coordinates in the average template space across participants in the age group. Figure 4 shows the group-level channel locations overlaid on the age-matched average templates. Lastly, we averaged the source–detector separation distances by age group. The devfOLD toolbox displays the average age group estimations of specificity values and channel properties.

### 2.10 Toolbox

The toolbox was developed in MATLAB2020a App Designer. The toolbox and estimations of all age groups are available in a GitHub repository: https://github.com/nirx/devfOLD. The toolbox was designed to display channel specificity and channel information (coordinates and separation distances) for (1) averaged age group estimations, (2) an individual participant, and (3) an average age-appropriate template. The estimations results were stored in MATLAB files (*.mat) organized in folders named by the (1) age name (e.g., 3Months), (2) a participant number (e.g., S0301), and (3) average template number for the given age group (e.g., S0300 for the 3-month age group). The variables that are stored in *.mat files are described in Table S3 in the Supplemental Material. A summary of the head model construction and channel-to-ROI specificity estimation steps is displayed in Fig. S1 in the Supplemental Material. We tested the devfOLD toolbox on all MATLAB OS platforms (Windows 2020b, Mac OSX 2020b, Linux 2020a, Windows, and Mac OSX MATLAB online). We presume that it works on prior MATLAB versions and will work on future ones.

## 3 Results

### 3.1 Toolbox

#### 3.1.1 Main interface

We developed the devfOLD toolbox to provide age-specific estimations of channel specificity and more extended selections of developmentally appropriate ROI parcellations. The example data for this paper contain averaged age group estimations for the 3-month, 6-month, and 20- to 24-year age groups (3Months, 6Months, and Adults), a 3-month-old individual participant (S0131), and a 3-month average template (S0300). We used six stereotaxic atlases constructed on individual participants.^8^^,^^27^^,^^47^ These include a lobar atlas identifying the major cortical lobes and some sublobar/subcortical regions, the Hammers atlas,^48^ Brainnetome connectivity atlas,^49^ the DKT atlas from FreeSurfer,^50^ the Infant FreeSurfer,^51^^,^^52^ the LONI Probabilistic Brain Atlas (LPBA40),^53^ and the AAL3 atlas that is implemented in the SPM12 software.^54^

**Fig. 5:**
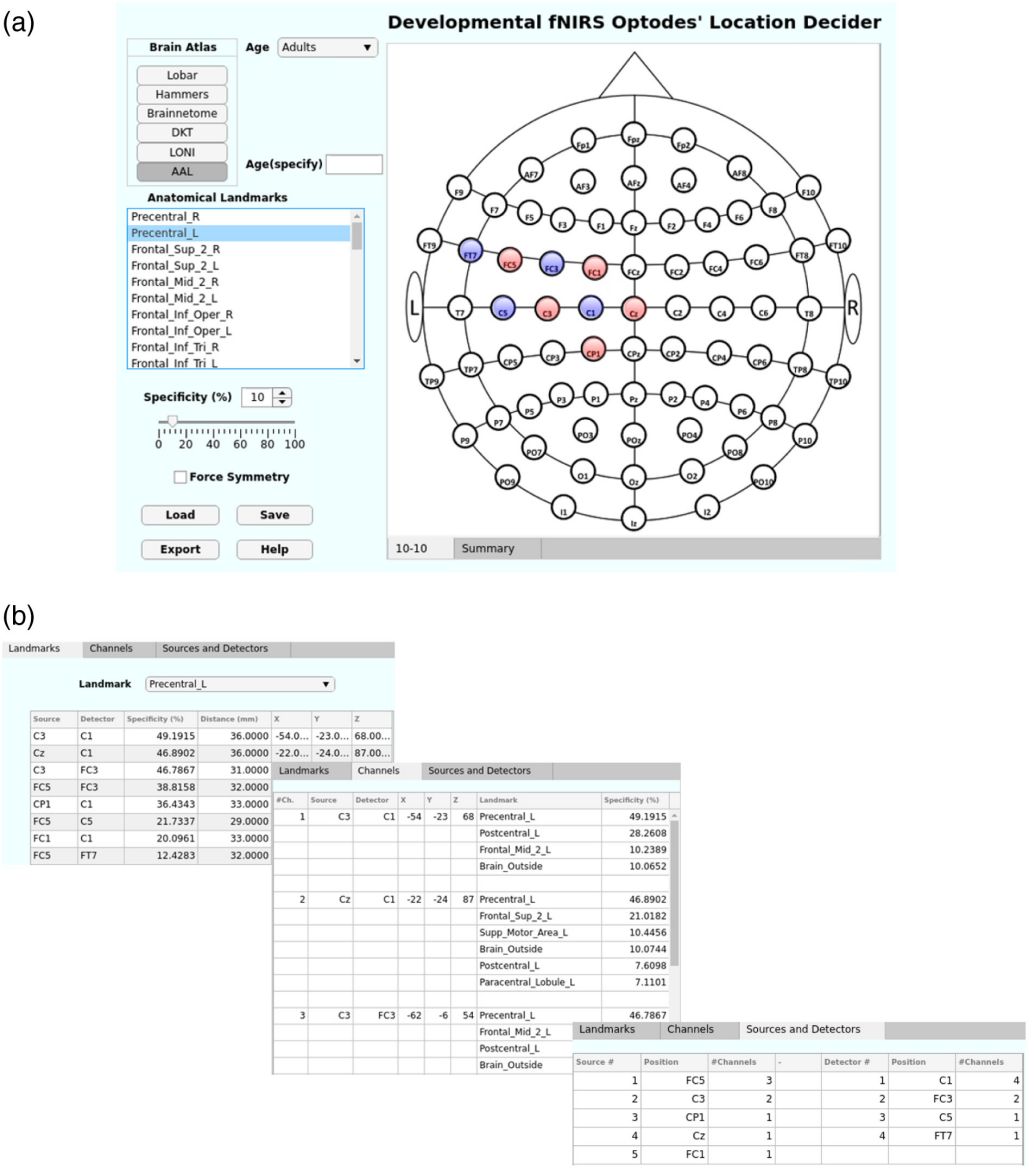
GUI of the developmental fNIRS Optodes” Location Decider, devfOLD toolbox. (a) Main GUI that displays channel positions for user-selected ROI. In the example, the “Precentral_L” ROI from the AAL3 atlas was selected with specificity (%) >10 for the “Adult” (20 to 24 years) age group. (b) Illustration of the content in the “Summary” tab for the ROI and specificity specification. Specificity and channel information are organized into the “Landmarks,” “Channels,” and “Sources and Detectors” subtabs.

The devfOLD graphical user interface (GUI) is shown in Fig. 5. Figure 5(a) shows an example of channels (with specificity >10%) that are sensitive to the left precentral gyrus (Precentral_L) from the AAL3 atlas for the “Adult” (20 to 24 years) age group. The user can also specify the data to be displayed in the “Age(specify)” box by typing in the data folder name (e.g., S0131) when the “Age” dropdown list is set to “Default.” The source (red) and detector (blue) locations are displayed once the user has specified an atlas from “Brain Atlas” and an ROI from “Anatomical Landmarks.” The specificity cut-off can be adjusted from the default value (30%) to the desired percentage (e.g., 10% in Fig. 5). The devfOLD toolbox displays all regions of a given atlas in “Anatomical Landmarks,” including noncortical/subcortical regions with <1% specificity. As observed in the fOLD toolbox, there was hemispheric asymmetry in channel-to-ROI specificity estimates. This could be attributed to anatomical differences between the two hemispheres in the scalp-to-cortex distances^8^^,^^61^ and channel sensitivity.^8^ The “Force Symmetry” option can be selected to force symmetric channel placement between the two hemispheres.

Figure 5(b) shows the corresponding content of the “Summary” tab for the “Precentral_L” selection. The “Summary” tab remains the same format as described in Ref. 9. The “Landmark” subtab displays the information of channels that are sensitive to the selected ROI. The “Channels” subtab displays all ROIs that can be measured by each of the selected channels with specificity greater than the user-specified cut-off. The user can use the information to evaluate the discriminability of the ROI. Channel C3-C1 shows good discriminability because it has high specificity to the ROI and relatively low specificity to other regions. The “Sources and Detectors” subtab lists the electrode/optode positions for the selected channels for measuring the ROI. The “Summary” data for all landmarks (ROIs) and channels are provided in age-specific look-up tables (3-0Months_MCX_10-10.xls, 6-0Months_MCX_10-10.xls, and 20-24Years_MCX_10-10.xls) in a GitHub repository.

#### 3.1.2 Additional age groups

The devfOLD toolbox can display estimations for additional age groups. We shared data on channel specificity and age-specific channel information (x,y,z coordinates and separation distances) computed for a wide range of age groups (2 weeks, 1 month, 2 months, 3 months to 12 months with 1.5-month age bins, 12 months to 24 months with 3-month age bins, 4 years, 12 years, and 20 to 24 years) in a GitHub repository. Users can copy the age-specific data folder to the “mat” folder where the toolbox data are stored. Estimates for the target age group will be displayed when the user enters the age name in the “Age(specify)” box as described above. We also provide the specificity estimates for the toolbox for a single 3-month-old participant (S0131). This is to show that the toolbox can also be used to display channel specificity and channel information estimated for subject-specific MRIs using the pipeline displayed in Fig. S1 in the Supplemental Material.

#### 3.1.3 Compatibility between devfOLD and fOLD toolbox

The devfOLD toolbox kept most of the fOLD toolbox features, with some important differences. The only common atlas shared between the devfOLD and fOLD toolbox is the LONI (LPBA40) atlas. The fOLD estimations of channel specificity to LPBA40 ROI parcellations for the MNI-ICBN152 atlas^22^ can be viewed by setting the “Age” option to “Default” and the “Brain Atlas” to “LONI” in the *devfOLD* toolbox. We eliminated the 10–5 extension considering the greater popularity of using the 10–10 system for channel placement and widely accepted algorithms for identifying 10–10 positions.^8^^,^^45^^,^^46^ We simulated 81 virtual electrode positions based on the “unambiguously illustrated 10–10 system.”^45^ Figure 2(a) shows the 10–10 electrode placement on an individual head MRI volume. Details for constructing the 10–10 locations are described in our previous studies.^8^^,^^46^ The “Mode” option was eliminated. We used individual MRI volumes to construct atlases and estimate specificity instead of using Colin27 or SPM12 templates. Hence, the devfOLD toolbox does not support the “Image Mask” Mode in the fOLD toolbox.

### 3.2 Age-Group Differences in Normalized Sensitivity

The age-related differences in the normalized sensitivity (normSens) are visible in the three example age groups. The voxelwise normSens value for a given channel represents a percentage of sensitivity relative to the whole MRI volume. Figure 3 shows a visualization of normSens for the channel FPz-AFz as an example. The channel sensitivity profile changes depending on the source–detector separation distance. The mean separation distance across the 130 channels for 3 months, 6 months, and 20 to 24 years was 23.7, 20.0, and 31.3 mm, respectively. In previous studies within the same sample, we found that the scalp-to-cortex distance was shorter in the 3-month and 6-month group than the adult group.^8^ Although the two infant groups had the comparable scalp-to-cortex distance, the sensitivity distribution extended deeper into the cortex in the 3-month than the 6-month group at 20 to 25 mm separation.^5^ This is consistent with the visualization displayed in Fig. 3. The sensitivity profiles for both the 6-month and the adult group were characterized by high peak in fluence value at shallow distances into the brain and steep decline as the light travels through the cortex.^5^ Given the adult group also has greater scalp-to-cortex distance, it is conceivable that greater fluence values are largely confined in the shallow regions in the adult compared to the 3-month head model, as shown in Fig. 3.

### 3.3 Differences in ROI-to-Channel Mapping between Head Models

**Fig. 6:**
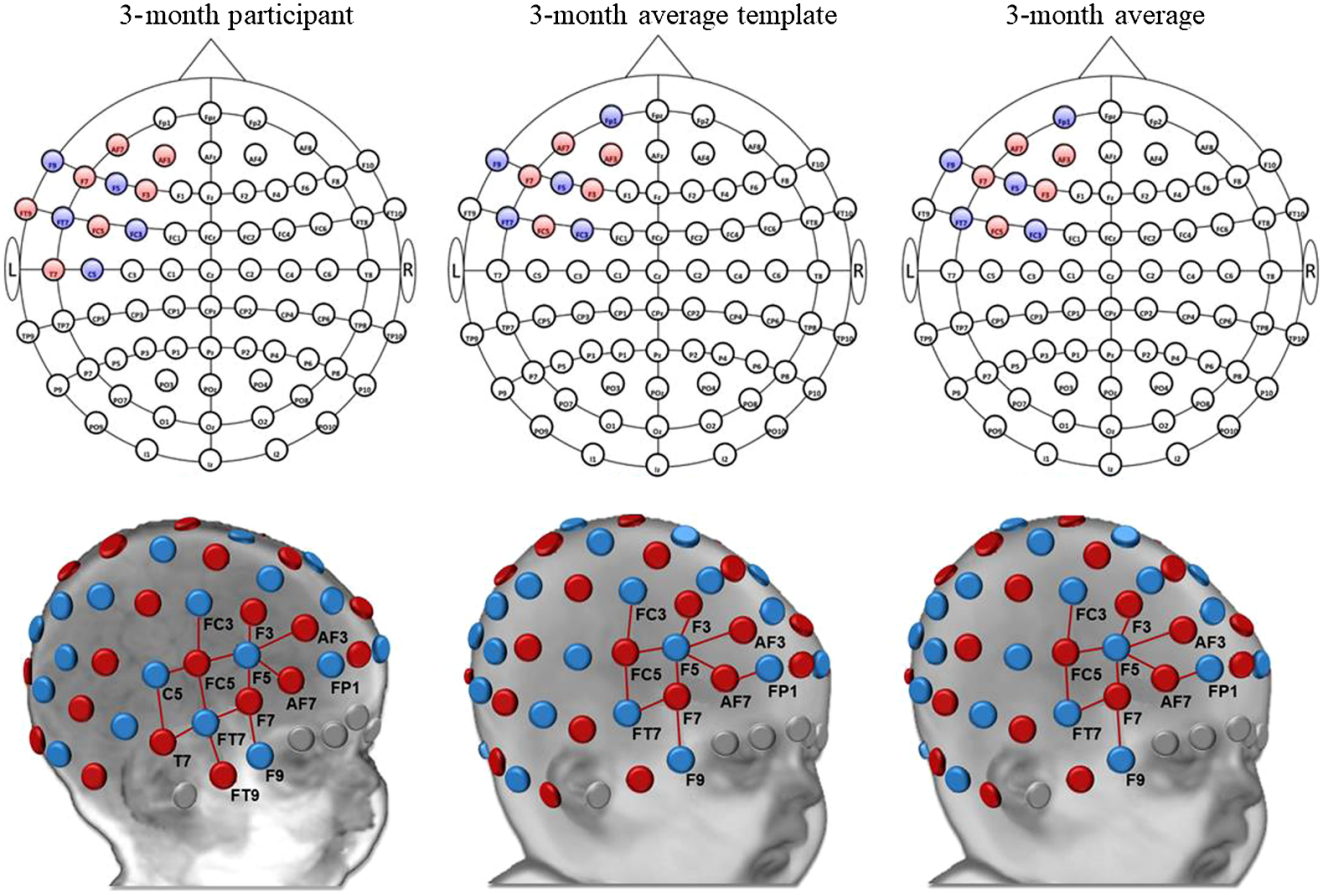
Source–detector channel configurations for the LIFG for 3 months of age. This is an example demonstrating that the devfOLD toolbox can be used to display channel placements estimated using an individual participant’s head model, an age-matched average template, or by averaging age group results computed from individual head models. For the 3-month participant and 3-month average template, specificity values were computed using a single input head model. For the 3-month average, the mean specificity value for each channel was calculated by averaging the sensitivity values for each ROI across participants at age 3-month (N=38). The mean value was then divided by the sum of averaged sensitivity for all ROIs. Detailed computation procedures are described in Sec. 2. The specificity threshold was set to 15%. The 10–10 maps above were generated by the devfOLD toolbox.

ROI-to-channel mapping showed both overlapping and nonoverlapping results when comparing estimations from different head models. The devfOLD toolbox provides specificity estimations for all ROI parcellations using a 3-month individual head model, a 3-month average template, and the 3-month group average. Figure 6 shows the channel configuration for the left inferior frontal gyrus (LIFG) from the LPBA40 atlas derived from the three types of estimations with the specificity threshold of 15%. The average template and age group average yield the same configuration. In contrast, additional channels, FC5-C5 (42% specificity), T7-FT7 (42%), T7-C5 (26%), and FT9-FT7 (17%), were also sensitive to the LIFG for the 3-month-old infant.

### 3.4 Age-Related Differences in ROI-to-Channel Mapping Based on Age-Group Averaged Specificity Estimations

**Fig. 7:**
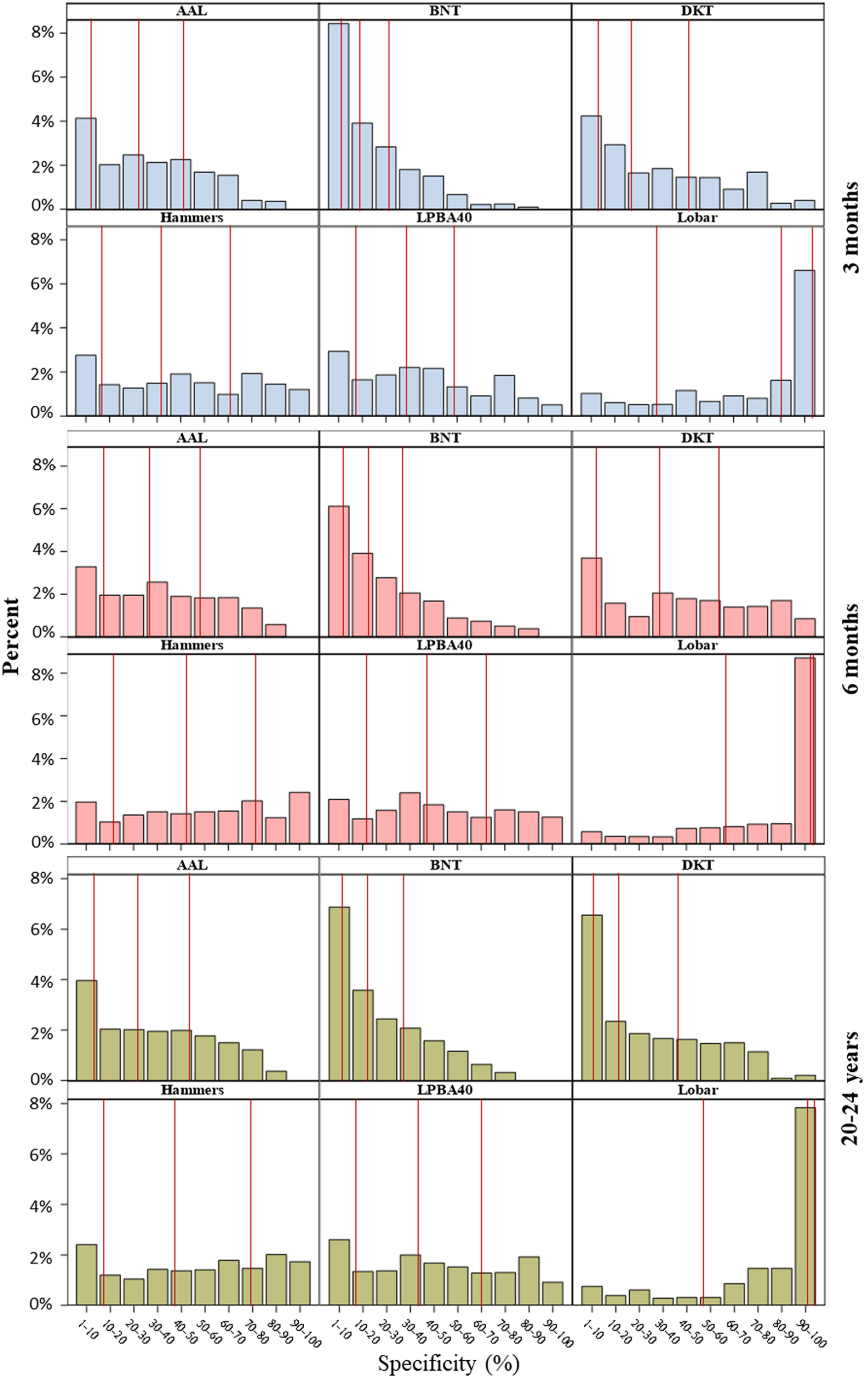
Distributions of channel specificity (%) by atlas types and age groups. Specificity values greater than 1% for channels sensitive to all ROI parcellations were included. Hence, a given channel had more than one specificity value given the one-to-many nature of ROI-to-channel correspondence. The red lines from the left to right mark the median, the 75th percentile, and the 90th percentile. Note: BNT, Brainnetome atlas; DKT, Desikan–Killiany–Tourville atlas.

The specificity estimations vary depending on the ROI and age group. Figure 7 shows the distributions of channel specificity (%) by atlas types and age groups. Specificity values greater than 1% for channels sensitive to all ROI parcellations were included. A given channel would have more than one specificity value given the one-to-many nature of ROI-to-channel correspondence. The red lines mark the median, 75th percentile, and 90th percentile. The shapes of the specificity distributions varied between atlas across all age groups. The specificity distribution within each atlas showed considerable consistency across age groups. The specificity clustered in large values (90% to 100%) for the Lobar atlas with larger ROIs but small values (1% to 10%) for atlases with smaller ROI parcellations (e.g., the Brainnetome and DKT) across age groups. This observation is consistent with our age-group averaged specificity computation where the channel specificity values to all detectable ROIs (in a specific atlas) are summed up to 100. Age differences were also visible in the Brainnetome and DKT atlas with smaller ROIs and the Lobar atlas with larger ROIs. The infant groups had a greater proportion of channels with small specificity (1% to 10%) for the Brainnetome ROIs. The adult group had a larger percentage of channels with small specificity for the DKT atlas. The 6-month and adult groups had greater proportions of channels with large specificity (90% to 100%) for the lobar atlas.

**Fig. 8:**
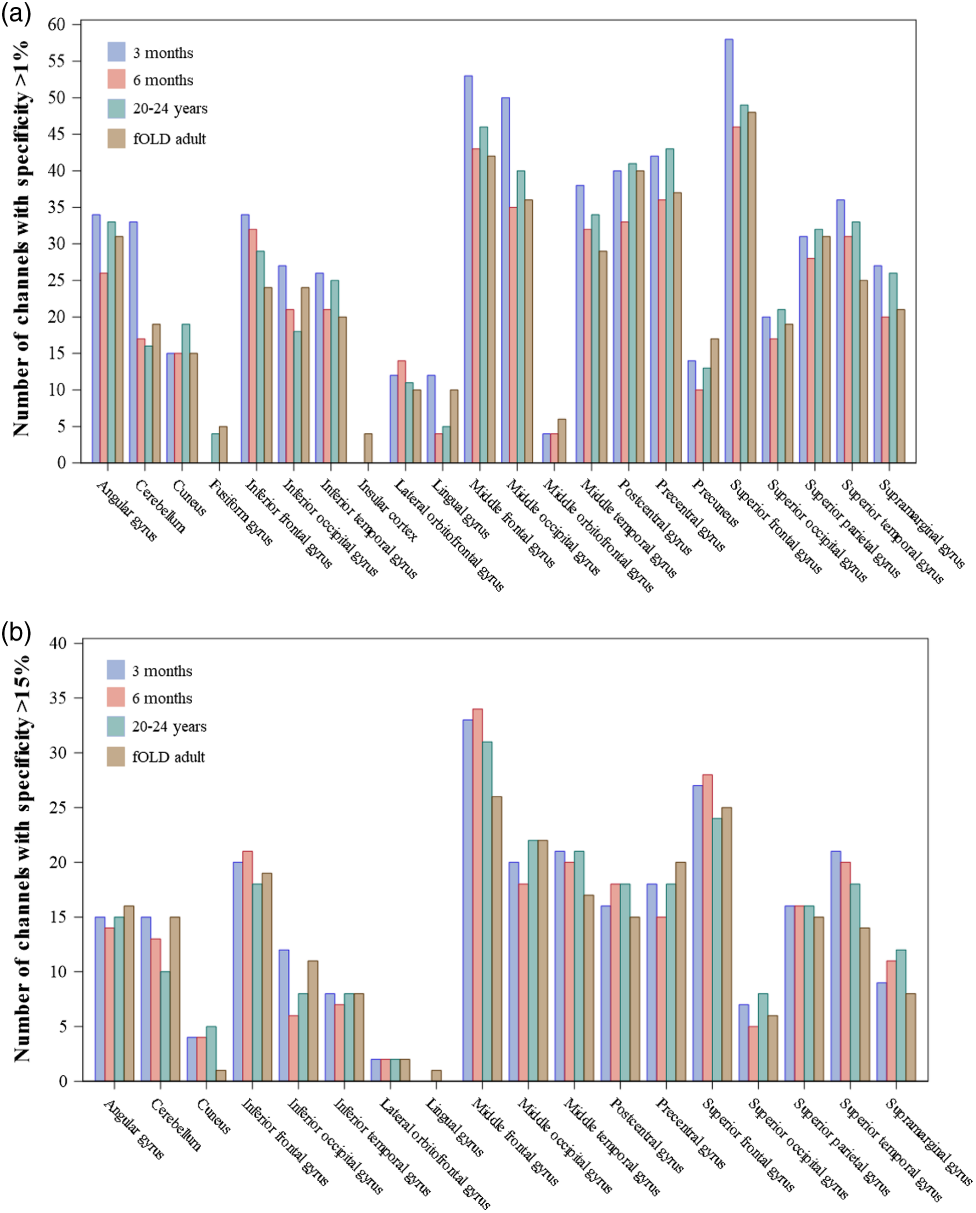
The number of channels sensitive to the brain ROIs in the LPBA40 atlas by age groups. The average age group specificity estimations for the 3-month, 6-month, and 20- to 24-year groups provided in the devfOLD toolbox are compared with the estimations from an adult template (SPM12) provided in the fOLD toolbox. Channels with specificity (a) greater than 1% and (b) 15% are included.

We examined differences in ROI-to-channel correspondence across the example age groups and the estimations from the fOLD adult template. ROIs from the LPBA40 atlas^53^ are considered as this is the only common atlas used in the fOLD and devfOLD toolbox. Figure 8 shows the number of channels that were sensitive to each of the LPBA40 ROIs by group. The specificity cut-off was set to 1% (upper panel) and 15% (lower panel), respectively. Figure 8 shows that the patterns of between-group differences changed with the user-specified specificity cut-off. For several ROIs (e.g., cerebellum, inferior frontal gyrus, middle temporal gyrus, and superior frontal gyrus), the number of channels that were sensitive to the ROI was greater for the 3-month group than the 20- to 24-year-old and fOLD-adult group at the 1% cut-off. The group differences between the 3-month and the adult groups were smaller at the 15% cut-off. The figure also reveals that the channel specificity to the gyrus rectus, parahippocampal gyrus, and cingulate was below 1%. The fusiform gyrus, insular cortex, lingual gyrus, middle orbitofrontal gyrus, and precuneus could only be measured with limited numbers of channels and with 1% specificity cut-off for all or most of the age groups. These ROIs have no specificity values with the 15% cutoff. This reflects the distance from the scalp optode recording to the ROIs and the inability of NIRS to measure deeper cortical areas. Increasing the cut-off to 15% also considerably reduced the number of channels mapped to the cuneus, lateral orbitofrontal gyrus, and superior occipital gyrus. Table S5A and S5B in the Supplemental Material present channel comparisons between pairs of the age groups at 1% and 15% specificity cut-off, respectively. The tables supplement Fig. 8 by showing the percentage of identical channels between the two groups for the LPBA40 ROIs out of all channels with specificity exceeded the cut-off for at least one of the age groups.

**Fig. 9:**
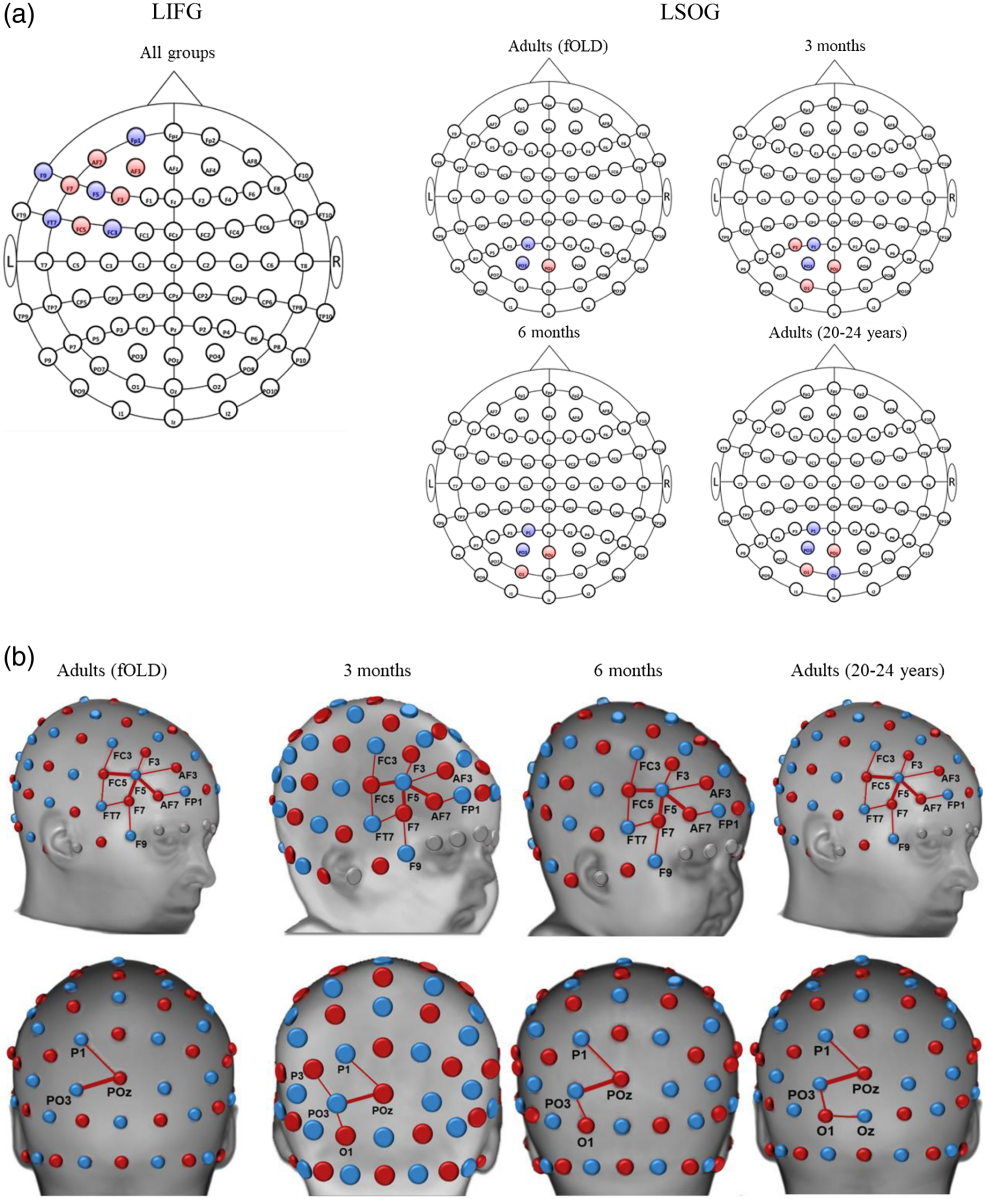
Source–detector channel configurations for the LIFG and the LSOG. The channel-to-ROI specificity for 3-month, 6-month, and 20- to 24-year age groups was estimated by averaging age group results computed from individual head models. The specificity threshold was set to 15%. (a) 2D displays of the channel arrangements from the devfOLD toolbox. The channel arrangement for the LIFG is identical for all groups. (b) 3D displays of the channel configurations for the LIFG (top row) and the LSOG (bottom row). Channels F7-F5, AF7-F5, FC5-F5 mapped to the LIFG and channel POz-PO3 mapped to the LSOG were bolded as they have specificity values greater than 50% for all age groups (also see Fig. 10). Channels are displayed on age-specific average brain templates.

**Fig. 10:**
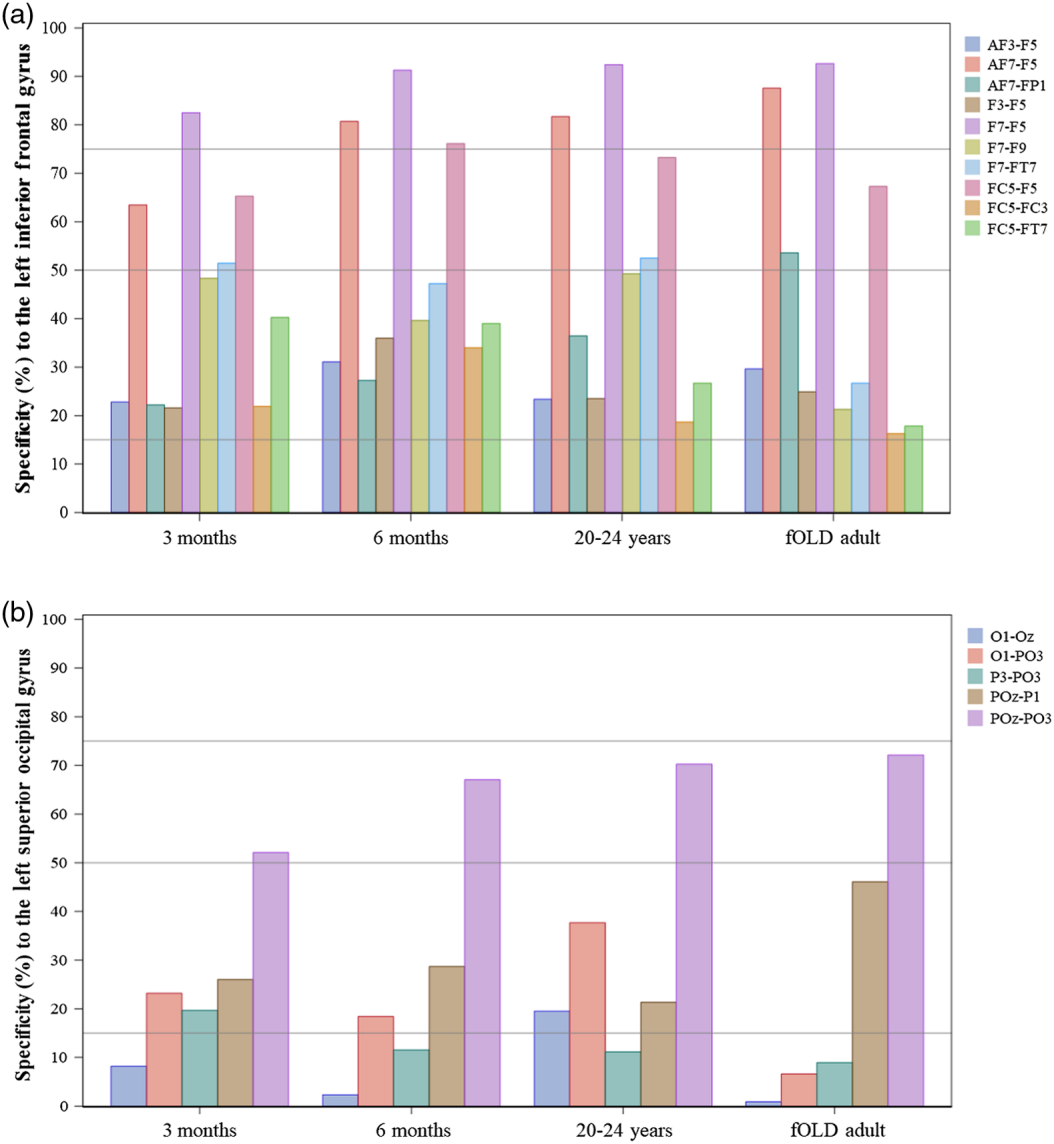
Specificity values for channels sensitive to (a) the LIFG and (b) LSOG by age groups (3 months, 6 months, 20 to 24 years, and fOLD adult template). Channels with specificity greater than 15% for at least one group were selected. The reference lines mark specificity at 15%, 50%, and 75%.

We selected two LPBA40 ROIs to further demonstrate between-group comparisons for ROI and specificity cut-off levels. Figure 9 shows comparisons among the original fOLD estimation using the SPM12 head template and our age-specific group averages (“3Months,” “6Months,” and “Adults”) for channels sensitive to the LPBA40 LIFG and the left superior occipital gyrus (LSOG). The specificity cut-off was set to 15%. The specificity values for these channels with specificity greater than 15% for at least one age group are shown in Fig. 10. The channel placements were identical across the adult template and the three averages for the LIFG. However, there were variations in channel specificity values across groups. For example, channel AF7-FP1 had specificity greater than 50% for the LIFG based on the fOLD estimation but its specificity was lower than 50% for the devfOLD groups. Channels F7-F5, AF7-F5, and FC5-F5 had specificity greater than 50% for all groups. The channel configurations for the LSOG were different across groups. Channel POz-PO3 had specificity greater than 50% for all groups, and the specificity for channel POz-P1 exceeded 15% but not 50% for all groups. Channel O1-PO3 was sensitive to this ROI for all the individual head models but not the SPM12 head model. Channel P3-PO3 was mapped to the LSOG only for the 3-month-olds, and channel O1-Oz was sensitive to this region only for the 20- to 24-year-olds.

## 4 Discussion

This study presents the devfOLD MATLAB toolbox. It is a user-friendly toolbox that facilitates the design of NIRS channel arrangements based on study-specific age groups and ROIs. The key improvement from the original fOLD toolbox^9^ is that the current version enables users to make decisions of channel placement based on channel-to-ROI specificity values computed using age-specific head models. The devfOLD toolbox is based on the computational methods used for the original fOLD toolbox and estimated channel specificity to developmentally appropriate ROI parcellations from six atlases for infant (2 weeks to 2 years with narrow age bins), child (4 and 12 years), and young adult age groups (20 to 24 years). The age-specific channel-to-ROI mapping can be displayed for an individual head model, an age-specific average template, or averaged estimations by age group (e.g., S0131, S0300, and 3Months in the devfOLD interface, respectively).

There were age-related differences in channel-to-ROI specificity. Figure 7 shows that the specificity values for ROI parcellations had different distributions depending on the atlas type and age group. The specificity values were computed from the voxelwise normalized sensitivity (normSens) values in both fOLD^9^ and devfOLD toolboxes. Figure 3 shows that the distribution of normalized sensitivity as the light traveled from the example channel location FPz-AFz into the cortex was visibly different among the 3-month, 6-month, and 20- to 24-year groups. This is consistent with the previous study with the same sample that revealed age-related differences in channel sensitivity profiles to the underlying cortex.^5^^,^^62^ The differences in sensitivity profiles between infant and adult groups can be attributed to morphological differences in the head and the brain. Infants have thinner extracerebral tissues (scalp and skull) and thicker CSF than adults,^4^ and the distance between the scalp and cortex was shorter in infants than in adults.^8^ Together, the existing evidence has underscored the importance of using age-specific head models for determining correspondence between scalp channel and cortical locations.

We further demonstrated that the between-age-group consistency and differences in channel configurations are driven by the cortical location and the user-specified specificity cut-off. Figure 8 compared the number of channels that were sensitive to LPBA40 ROIs across groups (3 months, 6 months, 20 to 24 years, and fOLD adult) at 1% and 15% specificity cut-off, respectively. Table S5A and S5B in the Supplemental Material additionally presented the percentage of identical channels for measuring each LPBA40 ROI between groups at the two cut-off values. Figures 9 and 10 provided between-group comparisons in channel configurations for the LPBA40 LIFG and LSOG as examples. At a lower specificity cut-off (15%), the channel configuration was the same across groups for the LIFG but considerably different for the LSOG. For both ROIs, there were differences in specificity values across groups at channels with specificity below 50%. Based on the visualizations, a researcher would select channel F7-F5, AF7-F5, and FC5-F5 to measure the LIFG, and channel POz-PO3 to measure the SOG if the goal is to have identical channel placement that can achieve high specificity across the infant and adult groups. Cai et al.^7^ also highlighted that cortical location affects age-related differences in channel-to-ROI mapping. They examined the brain regions with the highest sensitivity to each 10–10 channel location and found that about half of the channel’s locations corresponded to the same brain regions across 0-, 1-, and 2-year age groups. However, the channel locations along the longitudinal fissure showed the lowest consistency in channel-to-ROI correspondence across age groups. Hence, devfOLD and fOLD toolbox users need to make case-by-case decisions about the channel placement based on their ROIs and target age groups.

### 4.1 Applications for NIRS or fNIRS Studies

Our age-specific sensitivity estimations can be used to ensure that the channel-to-ROI specificity values are comparable across age groups in cross-sectional or longitudinal studies with developmental samples. The specificity cut-off is a free parameter. We have demonstrated that the specificity cut-off setting influences the coverage of the ROI (i.e., the number of channels mapped to the ROI) and the consistency in channel placement between age groups. We recommend devfOLD (or fOLD) toolbox users generate similar visualizations as Fig. 10 and summary statistics in Table S5 in the Supplemental Material to explore age-group differences in ROI-to-channel mapping and determine the appropriate cut-off based on their selected ROI(s), targeted age group(s), and the number of available source and detector optodes. The visualizations can be performed using data from the Excel look-up tables available in a GitHub repository: https://github.com/nirx/devfOLD/tree/master/Look-up%20Tables. A higher specificity cut-off could help safeguard between-age-group consistency in the channel configuration. The selectivity also needs to be balanced with sufficient coverage for the selected ROI(s).

The devfOLD toolbox allows for flexibility of using different head models to estimate channel-to-ROI correspondence. Quantifying the sensitivity of NIRS channels to detecting changes in hemoglobin concentration of a given ROI is a fundamental step for optimizing channel placement. Accurate channel-to-ROI sensitivity estimations for individual participants require head models constructed from participants’ own structural MRIs. The subject-specific approach has been implemented in existing methods for channel arrangement optimization.^17^^,^^20^ The devfOLD toolbox allows for the subject-specific implementation, as it can display channel specificity estimates from an individual participant. However, this optimization method is time-consuming, requires existing structural MRIs from the study participants, and limits the application for developmental NIRS studies where collecting structural MRIs from infants and young children is challenging. This study has illustrated that an alternative to using subject-specific MRIs is using age-matched average templates or individual head models from a shared database (e.g., Neurodevelopmental MRI Database).^26^^–^^31^ We recommend using the averaged specificity estimates by age groups for standalone NIRS studies that require researchers to design the channel configuration prior to data collection. Compared to using a single template such as those used in the fOLD toolbox^9^ and Array Designer,^16^ the group averaged estimates minimize errors caused by individual anatomical differences,^14^ thus provide more accurate quantification of channel-to-ROI correspondence for the target age group.

The devfOLD toolbox informs subsequent NIRS localization procedures and facilitates the implementation of best practices for NIRS research. The estimation of channel-to-ROI sensitivity is also a crucial step for subsequent image reconstruction that localizes the neural activities from the channel space into specific cortical regions.^63^^,^^64^ For standalone NIRS studies in developmental samples, age-matched average templates from the Neurodevelopmental MRI Database have been used for image source inverse reconstruction.^21^^,^^63^^,^^65^ It is a less complicated method comparing to using individual head models from the database that are closely matched to individual study participants’ age and head size. Our inclusion of average templates and age-group averages of specificity estimates in the devfOLD toolbox enables researchers to compare between the two possible “substitutes” to determine whether there is an advantage to use the more complicated method for image reconstruction based on the study-specific age group(s) and ROI(s). In addition, the recent guide for “best practices for fNIRS publications”^66^ emphasized the importance of reporting NIRS array configuration and the channel sensitivity profiles to underlying ROIs. Hence, the devfOLD toolbox allows for *a prior* determination of optode placement based on channel-to-ROI sensitivity also contributes to enhancing standardization and reproducibility of NIRS research.

### 4.2 Limitations

The devfOLD toolbox has some limitations comparing to existing channel placement optimizers.^16^^,^^17^ First, the toolbox has a highly constrained solution space that considers only 130 channels with predefined sources and detectors on neighboring 10–10 positions. The toolbox facilitates age-specific selections of channels in the 10–10 system with high specificity to user-specified ROIs. However, it cannot be used as an “optimization” tool to find the optimal channel configuration for a given ROI as other existing tools.^16^^,^^17^^,^^18^^,^^20^ The constrained pool of source–detector combinations also means that the source–detector separation distances are fixed. The average distance was 23.7 mm (SD=3.3) for the 3-month group, 20.0 mm (SD=3.7) for the 6-month-olds, and 31.3 mm (SD=3.9) for the adult group. These are within the distance range of conventional channel placement for infants 20 to 30 mm^67^ and adults 30 to 35 mm.^68^ However, we know that the channel sensitivity profile changes as a function of separation distances.^6^ As the distances increase, the sensitivity distribution extends deeper into the brain and the fluence strength decreases. Furthermore, there are important age differences in how sensitivity profile change with varying separation distances.^5^^,^^62^ Therefore, the separation distance should be optimized based on the ROI and age group. A related limitation is that the recommended channel configuration obtained from the toolbox may require manual adjustment depending on the participant’s head size and shape. The “Summary” tab displays the separation distance for each channel based on estimations from the age-matched average template. We recommend researchers test the preliminary configuration with pilot infant or child participants to ensure the placement and source–detector separation distances are appropriate.

The solution space of potential channel positions can be increased using denser optode systems. The Array Designer^16^ estimated S-D channel DOT sensitivity for 77,995 channels formed by source–detector pairings from the 10–2.5 system.^69^ The toolbox allows users to specify ROI, channel separation range, and available optodes. An algorithm was applied to optimize ROI coverage and total sensitivity of the channel configuration. Fu and Richards^5^ selected channels with separation distances ranged from 10 to 60 mm with 5-mm increments from a pool of 63,903 source–detector pairings from the 10–5 system.^45^ It is important to note that, due to head-size differences across age groups, the number of potential channels varied with target separation distance and age group.^5^ This issue increased the complication of optimizing sensitivity and ROI coverage for age-specific channel configuration.

It is also important that future age-specific toolboxes provide specificity estimates of multiple channels to a given ROI (i.e., solve the combinatorial problem^16^). The devfOLD toolbox provides specificity estimates for the one-to-many channel-to-ROI mappings. However, it does not show sensitivity estimates of a given channel configuration to an ROI. Hence, users cannot conduct within-age-group or between-age-group comparisons between channel configurations that contain channels with specificity values that exceeded the specified cut-off. This constraint underscores the importance of carefully exploring specificity data for the ROI(s) and targeted age group(s) to determine an appropriate specificity cut-off. Further toolbox development is needed to facilitate the assessment of whether a given channel configuration can achieve the same level of sensitivity to one or multiple ROIs across age groups.

## 5 Conclusions

The devfOLD and the fOLD toolbox highlight the importance of incorporating age-specific head models for optimizing channel arrangements. The devfOLD toolbox provides specificity values and channel properties obtained from age-specific average templates, averaged age group estimations, and individual head models. It has important utilities for standalone NIRS studies where the participants’ own MRIs are not collected. Our findings suggest that future optimization tools should allow for user-defined head models. The selection can be an individual head model constructed from a participant’s MRI, a study-specific average template, a publicly shared age-matched average template, or a group of publicly shared, age-matched head models. A future direction for the toolbox development is to provide age-specific specificity estimates of a given channel configuration to selected ROI(s).

## Code, Data, and Materials Statement

The age-specific average templates, including T1, DOT sensitivity average template can be accessed from the “Neurodevelopmental MRI Database.” The database is available online: https://jerlab.sc.edu/projects/neurodevelopmental-mri-database/ and https://www.nitrc.org/projects/neurodevdata/ for access. Details on the online access are provided in Ref. 29. The devfOLD toolbox and associated data are accessible in a GitHub repository: https://github.com/nirx/devfOLD.
