# Supplementary material for: devfOLD: a toolbox for designing age-specific fNIRS channel placement
Source: Neurophotonics. 2021 Dec 6;8(4):045003. doi: 10.1117/1.NPh.8.4.045003 (PMC8647945; doi:10.1117/1.NPh.8.4.045003)
Supplement: Supplementary file 1 [file NPh_008_045003_SD001.pdf]

Supplemental Table 1. Demographical information of the full study participants by age group, sex, and data source. The current study presents estimations for the 3-month, 6-month, and 20-to-24-year groups as examples.
Note. ABIDE=Autism Brain Imaging Data Exchange; BCP=Baby Connectome Project; EBDS=Early Brain Development Study; IBIS=Infant Brain Imaging Study; MCBI=McCausland Center for Brain Imaging; PING=Pediatric Imaging, Neurocognition, and Genetics Data Repository

**Participant Information**			**Data Source**					
**Age Group**	**Total *N***	**Female *N***	**ABIDE *N***	**BCP *N***	**EBDS *N***	**IBIS *N***	**MCBI and Collaboration Sites *N***	**PING *N***
2 Weeks	41	24	0	3	38	0	0	0
1 Month	96	40	0	17	79	0	0	0
2 Months	68	40	0	8	60	0	0	0
3 Months	38	21	0	24	0	0	14	0
4.5 Months	54	29	0	41	0	0	13	0
6 Months	74	35	0	0	0	60	14	0
7.5 Months	61	17	0	0	0	49	12	0
9 Months	60	35	0	48	0	3	9	0
10.5 Months	42	21	0	40	0	0	2	0
12 Months	109	36	0	0	0	89	20	0
15 Months	78	41	0	63	0	8	7	0
18 Months	76	31	0	36	0	8	32	0
2 Years	66	22	0	0	0	65	1	0
4 Years	24	9	0	0	0	0	10	14
12 Years	37	14	9	0	0	0	28	0
20-24 Years	134	77	0	0	0	0	134	0

Supplemental Table 2. Source-detector combinations of the 130 channels.

Number	Channel Name	Source	Detector
1	FPZ-FP1	FPZ	FP1
2	FPZ-AFZ	FPZ	AFZ
3	FPZ-FP2	FPZ	FP2
4	AF3-FP1	AF3	FP1
5	AF3-AFZ	AF3	AFZ
6	AF3-F1	AF3	F1
7	AF3-F5	AF3	F5
8	AF7-FP1	AF7	FP1
9	AF7-F5	AF7	F5
10	FZ-AFZ	FZ	AFZ
11	FZ-F1	FZ	F1
12	FZ-FCZ	FZ	FCZ
13	FZ-F2	FZ	F2
14	F3-F1	F3	F1
15	F3-F5	F3	F5
16	F3-FC3	F3	FC3
17	F7-F5	F7	F5
18	F7-FT7	F7	FT7
19	F7-F9	F7	F9
20	FC1-F1	FC1	F1
21	FC1-FC3	FC1	FC3
22	FC1-C1	FC1	C1
23	FC1-FCZ	FC1	FCZ
24	FC5-F5	FC5	F5
25	FC5-FC3	FC5	FC3
26	FC5-FT7	FC5	FT7
27	FC5-C5	FC5	C5
28	C3-FC3	C3	FC3
29	C3-C1	C3	C1
30	C3-C5	C3	C5
31	C3-CP3	C3	CP3
32	T7-FT7	T7	FT7
33	T7-C5	T7	C5
34	T7-TP7	T7	TP7
35	CP1-C1	CP1	C1
36	CP1-CP3	CP1	CP3
37	CP1-P1	CP1	P1
38	CP1-CPZ	CP1	CPZ
39	CP5-C5	CP5	C5
40	CP5-CP3	CP5	CP3

41	CP5-TP7	CP5	TP7
42	CP5-P5	CP5	P5
43	P3-CP3	P3	CP3
44	P3-P1	P3	P1
45	P3-P5	P3	P5
46	P3-PO3	P3	PO3
47	P7-TP7	P7	TP7
48	P7-P5	P7	P5
49	P7-PO7	P7	PO7
50	P7-P9	P7	P9
51	O1-PO3	O1	PO3
52	O1-PO7	O1	PO7
53	O1-I1	O1	I1
54	O1-OZ	O1	OZ
55	PO9-PO7	PO9	PO7
56	PO9-I1	PO9	I1
57	PO9-P9	PO9	P9
58	FT9-FT7	FT9	FT7
59	FT9-F9	FT9	F9
60	TP9-TP7	TP9	TP7
61	TP9-P9	TP9	P9
62	CZ-C1	CZ	C1
63	CZ-FCZ	CZ	FCZ
64	CZ-CPZ	CZ	CPZ
65	CZ-C2	CZ	C2
66	PZ-P1	PZ	P1
67	PZ-CPZ	PZ	CPZ
68	PZ-P2	PZ	P2
69	POZ-P1	POZ	P1
70	POZ-PO3	POZ	PO3
71	POZ-OZ	POZ	OZ
72	POZ-P2	POZ	P2
73	POZ-PO4	POZ	PO4
74	IZ-I2	IZ	I2
75	IZ-I1	IZ	I1
76	IZ-OZ	IZ	OZ
77	AF4-AFZ	AF4	AFZ
78	AF4-FP2	AF4	FP2
79	AF4-F2	AF4	F2
80	AF4-F6	AF4	F6
81	AF8-FP2	AF8	FP2
82	AF8-F6	AF8	F6
83	F4-F2	F4	F2

84	F4-F6	F4	F6
85	F4-FC4	F4	FC4
86	F8-F6	F8	F6
87	F8-F10	F8	F10
88	F8-FT8	F8	FT8
89	FC2-FCZ	FC2	FCZ
90	FC2-F2	FC2	F2
91	FC2-FC4	FC2	FC4
92	FC2-C2	FC2	C2
93	FC6-F6	FC6	F6
94	FC6-FC4	FC6	FC4
95	FC6-FT8	FC6	FT8
96	FC6-C6	FC6	C6
97	FT10-F10	FT10	F10
98	FT10-FT8	FT10	FT8
99	C4-FC4	C4	FC4
100	C4-C2	C4	C2
101	C4-C6	C4	C6
102	C4-CP4	C4	CP4
103	T8-FT8	T8	FT8
104	T8-C6	T8	C6
105	T8-TP8	T8	TP8
106	CP2-CPZ	CP2	CPZ
107	CP2-C2	CP2	C2
108	CP2-CP4	CP2	CP4
109	CP2-P2	CP2	P2
110	CP6-C6	CP6	C6
111	CP6-CP4	CP6	CP4
112	CP6-TP8	CP6	TP8
113	CP6-P6	CP6	P6
114	TP10-TP8	TP10	TP8
115	TP10-P10	TP10	P10
116	P4-CP4	P4	CP4
117	P4-P2	P4	P2
118	P4-P6	P4	P6
119	P4-PO4	P4	PO4
120	P8-TP8	P8	TP8
121	P8-P6	P8	P6
122	P8-P10	P8	P10
123	P8-PO8	P8	PO8
124	O2-I2	O2	I2
125	O2-OZ	O2	OZ
126	O2-PO4	O2	PO4

127	O2-PO8	O2	PO8
128	PO10-I2	PO10	I2
129	PO10-P10	PO10	P10
130	PO10-PO8	PO10	PO8

Supplemental Table 3. Optical properties used in Monte Carlo photon migration simulations. μ_a is the optical absorption coefficient; μ_s is the scattering coefficient, *g* is the anisotropy coefficient, and *N* is the index of refraction.

Tissue Type	μ_a (mm^{-1})	μ_s (mm^{-1})	*g*	*N*
White matter	0.07	40.1	0.85	1.37
Gray matter	0.02	8.4	0.90	1.37
CSF	0.0004	1	0.99	1.37
Dura	0.0101	80	0.99	1.37
Skull	0.0101	100	0.99	1.37
Skin	0.0101	80	0.99	1.37
Muscle	0.0101	80	0.99	1.37
Eyes	0.0004	1	0.99	1.37
Nasal Cavity	0.0101	80	0.99	1.37
Non-myelinated Axons	0.07	40.1	0.85	1.37

Supplemental Table 4. Descriptions of variables stored in .mat files for the *devfOLD* toolbox.

Directory	File Name	Variable	Description
../Developer/mat/10-10[1]	channels1010.mat	labels	Source-detector combinations of the 130 channels
	labels1010.mat	detectors	Detector optodes
		sources	Sources optodes
../Developer/mat/(FolderName)[2]	(FolderName)MCX1010.mat	ch_coord	Coordinates (x,y,z) for all channel locations (in individual head model space or age-matched average template space)
		chdist	Source-detector channel separations (mm) for all channels
		det	Coordinates (x,y,z) for the detector optode locations (in individual head model space or age-matched average template space)
		src	Coordinates (x,y,z) for the source optode locations (in individual head model space or age-matched average template space)
	(AtlasName)1010_(AgeName).mat[3]	acc_brain	For each channel, sensitivity to brain regions of interest (ROIs) and "Brain_Outside"
		accuracy	For each channel, sensitivity to non-brain segments (Scalp, Skull, and CSF), ROIs, and "Brain_Outside"
		anatom_brain	The ROI labels corresponding to "acc_brain" (plus "Brain_Outside")
		anatom_label	The labels corresponding to "accuracy"
		Label	ROI labels of the atlas (n*1 cell)
		landmarks	ROI labels of the atlas (1*n cell)
		roi	Channel-to-ROI specificity for each ROI in the atlas.<br>roi{n}.landmark: label of the ROI<br>roi{n}.channels: the channel number of each channel that can measure the ROI (corresponding to roi{n}.perc)<br>roi{n}.labels: labels of the source-detector combination of each channel that can measure the ROI (corresponding to roi{n}.perc)

		roi{n}.perc: specificity value (in descending order) of each channel that can measure the ROI
	template	The string is ‘(AgeName)MCX’

Notes:

1. The rest of the *.mat files in the directory contain data from the fOLD toolbox. They are kept as place holder files for the *devfOLD* toolbox.
2. If the folder has is an age name (e.g. “3Months”), it contains averaged age estimations. Folder “S0301” contains estimations from an example participant. Folder “S0300” contains estimations obtained using the age-matched average templates for the 3-month age group). To display the information in the *devfOLD* toolbox, the user enters the folder name into the “Age (specify)” box.
3. There are 6 (AtlasName)1010_(AgeName).mat files in total, one for each atlas.

Supplemental Table 5A. The percentage of overlapping channels that are sensitive to the LPBA40 regions of interest (ROIs). Channels with specificity greater than 1% were included. The shaded cells indicate that there is no channel sensitive to the ROI in neither of the age group.

Region of Interest	3 Months vs. 6 Months	3 Months vs. 20-24 Years	3 Months vs. fOLD Adult	6 Months vs. 20-24 Years	6 Months vs. fOLD Adult	20-24 Years vs. fOLD Adult
Superior frontal gyrus	79.31	75.41	70.97	93.88	88.00	83.02
Middle frontal gyrus	81.13	80.00	79.25	93.48	93.18	91.30
Inferior frontal gyrus	88.57	85.29	70.59	84.85	75.00	82.76
Precentral gyrus	85.71	77.08	88.10	79.55	82.50	73.91
Middle orbitofrontal gyrus	60.00	0.00	25.00	0.00	42.86	0.00
Lateral orbitofrontal gyrus	73.33	53.33	46.67	66.67	60.00	90.91
Postcentral gyrus	78.05	88.37	77.78	80.49	73.81	80.00
Superior parietal gyrus	68.57	85.29	72.22	81.82	73.53	75.00
Supramarginal gyrus	74.07	70.97	65.52	76.92	70.83	80.77
Angular gyrus	71.43	63.41	62.50	68.57	78.13	64.10
Precuneus	71.43	92.86	63.16	76.92	58.82	57.89
Superior occipital gyrus	76.19	64.00	69.57	58.33	71.43	48.15
Middle occipital gyrus	70.00	60.71	62.26	82.93	73.17	72.73
Inferior occipital gyrus	71.43	66.67	75.86	85.71	66.67	61.54
Cuneus	100.00	78.95	66.67	78.95	66.67	61.90
Superior temporal gyrus	86.11	81.58	69.44	88.24	80.65	75.76
Middle temporal gyrus	84.21	75.61	76.32	88.57	84.85	80.00
Inferior temporal gyrus	80.77	96.15	76.92	84.00	95.24	80.00
Lingual gyrus	33.33	41.67	57.14	80.00	27.27	25.00
Fusiform gyrus		0.00	00.00	0.00	0.00	50
Insula cortex			0.00		0.00	0.00
Cerebellum	51.52	48.48	57.58	94.12	89.47	84.21

Supplemental Table 5B. The percentage of overlapping channels that are sensitive to the LPBA40 regions of interest (ROIs). Channels with specificity greater than 15% were included. The shaded cells indicate that there is no channel sensitive to the ROI in neither of the age group.

Region of Interest	3 Months vs. 6 Months	3 Months vs. 20-24 Years	3 Months vs. fOLD Adult	6 Months vs. 20-24 Years	6 Months vs. fOLD Adult	20-24 Years vs. fOLD Adult
Superior frontal gyrus	77.42	88.89	79.31	85.71	82.76	81.48
Middle frontal gyrus	91.43	93.94	78.79	91.18	76.47	83.87
Inferior frontal gyrus	95.24	90.00	95.00	85.71	90.48	85.00
Precentral gyrus	83.33	100.00	80.95	83.33	75.00	80.95
Middle orbitofrontal gyrus						
Lateral orbitofrontal gyrus	100.00	100.00	100.00	100.00	100.00	100.00
Postcentral gyrus	78.95	78.95	63.16	80.00	57.14	65.00
Superior parietal gyrus	100.00	100.00	93.75	100.00	93.75	93.75
Supramarginal gyrus	81.82	61.54	54.55	76.92	58.33	66.67
Angular gyrus	93.33	87.50	55.00	93.33	50.00	55.00
Precuneus						
Superior occipital gyrus	71.43	66.67	44.44	62.50	57.14	40.00
Middle occipital gyrus	90.00	61.54	68.00	66.67	60.00	51.72
Inferior occipital gyrus	50.00	66.67	53.33	75.00	30.77	26.67
Cuneus	60.00	28.57	25.00	50.00	25.00	20.00
Superior temporal gyrus	78.26	77.27	52.17	90.00	70.00	68.42
Middle temporal gyrus	95.24	82.61	80.95	86.36	85.00	80.95
Inferior temporal gyrus	87.50	45.45	60.00	50.00	66.67	60.00
Lingual gyrus			0.00		0.00	0.00
Fusiform gyrus						
Insula cortex						
Cerebellum	75.00	66.67	87.50	76.92	75.00	66.67

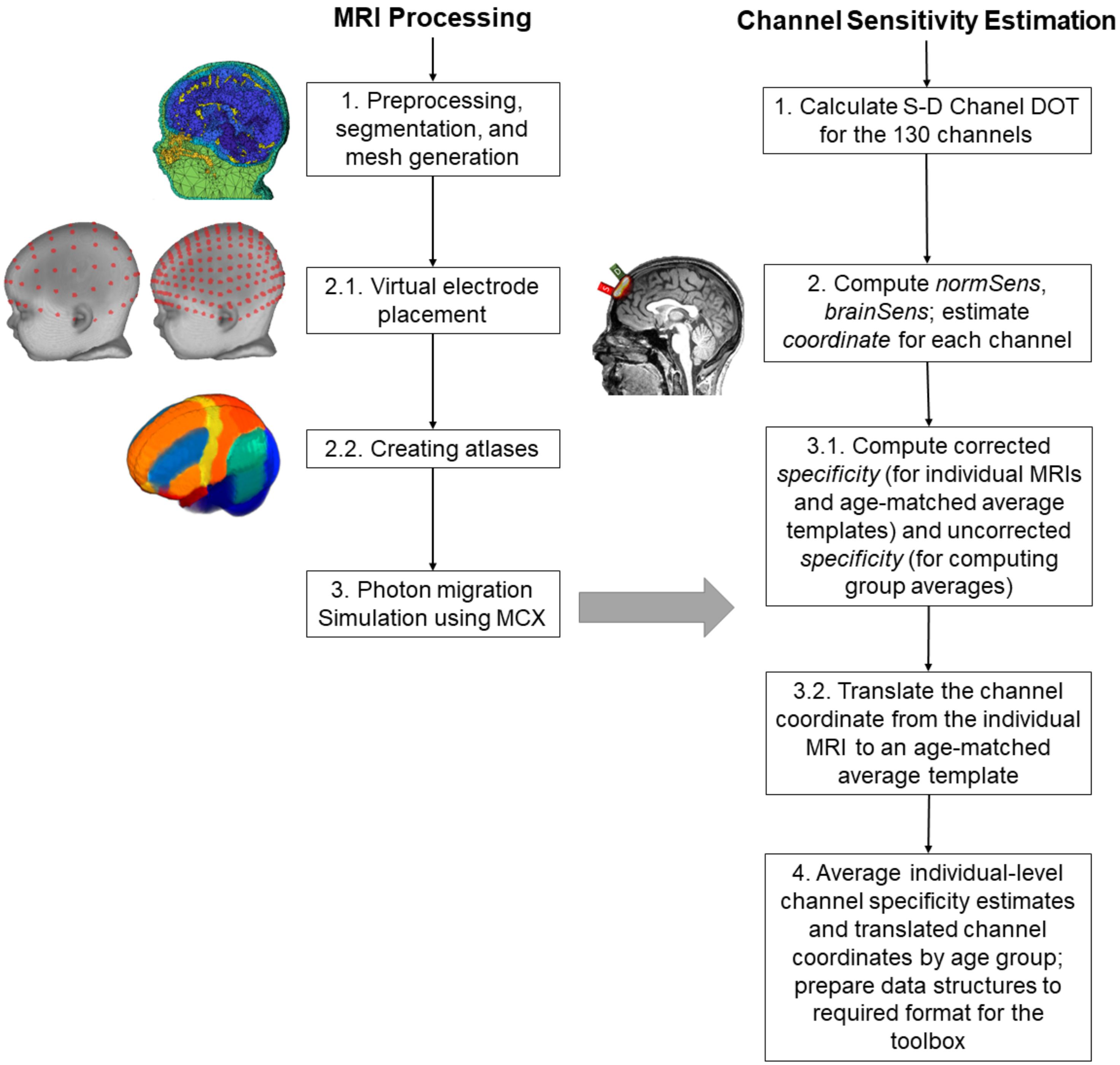

Fig S1. Pipeline for head model construction and channel sensitivity estimation. The procedures are detailed in the Methods of the Main Text. Each step is implemented in a separate MATLAB program.
